# Analysis of DNA-damage response to ionizing radiation in serum-shock synchronized human fibroblasts

**DOI:** 10.1007/s10565-017-9394-9

**Published:** 2017-05-03

**Authors:** Samantha Corrà, Riccardo Salvadori, Leonardo Bee, Vito Barbieri, Maddalena Mognato

**Affiliations:** 1 Department of Biology, School of Sciences, University of Padova, via U. Bassi 58 B, 35131 Padova, Italy; 2 Menarini Silicon Biosystems, 10355 Science Center Dr #210, San Diego, CA 92121 USA; 3 Department of Surgical, Oncological and Gastroenteric Sciences, University of Padova, via Giustiniani 2, Padova, Italy

**Keywords:** DNA-damage response, Gamma rays, Circadian clock, Human fibroblasts

## Abstract

Many aspects of cellular physiology, including cellular response to genotoxic stress, are related to the circadian rhythmicity induced by the molecular clock. The current study investigated if the cellular response to DNA damage is in relation to endogenous expression levels of the PER2 protein, a key component of the molecular regulatory system that confers rhythmicity in mammalian cells. Human normal fibroblasts (CCD-34Lu) were subjected to serum shock to induce circadian oscillations of the PER2 protein and then irradiated with γ- rays at times corresponding to the trough and peak expression of the PER2 protein. To better examine cellular response to DNA damage, the experiments performed in this study were carried out in non-proliferating CCD-34Lu fibroblasts in order to maintain the cell and circadian cycles separated while they were being exposed to genotoxic stress. Study results demonstrated that clonogenic cell survival, double-strand break repair kinetics, and TP53 protein levels were affected in the cells irradiated at the trough than in those irradiated at peak expression of the PER2 protein.

**Electronic supplementary material:**

The online version of this article (doi:10.1007/s10565-017-9394-9) contains supplementary material, which is available to authorized users.

## Introduction

Mammalian cells possess a cell-autonomous molecular clock which controls the timing of physiological processes and hence the cellular response to external stimuli, including genotoxic stress. All organisms adapt to daily environmental changes by synchronizing multiple molecular, biochemical, physiological, and behavioral processes through daily oscillations in biological processes that are controlled by an endogenous biochemical pacemaker that is termed the circadian clock (Savvidis and Koutsilieris 2012). The master clock is located in the hypothalamic suprachiasmatic nucleus (SCN), a small brain region containing 10,000–15,000 neurons. Core components of the mammalian circadian molecular clock, CLOCK (Circadian Locomotor Output Cycles Kaput) and BMAL1 (brain muscle Arnt-like 1), which are members of the bHLH-PAS transcription factor family, form a heterodimer, which binds to the E-box cis-regulatory enhancer elements of their target genes, including Period (PER) and Cryptochromes (CRYs). A negative feedback loop is achieved when the PERs and CRYs form heterocomplexes that translocate back to the nucleus and inhibit their own transcription. In addition to the primary feedback loops, another regulatory one, the “stabilizing loop” (Kwon et al. 2011), is formed by REV-ERBα and RORα, which are orphan nuclear receptors. REV-ERBα competes in the nucleus with RORα for binding to the ROR-responsive element (RORE) in the Bmal1 promoter (Gallego and Virshup 2007). While RORα activates Bmal1 transcription, REV-ERBα represses it. As a result, the cyclic expression of Bmal1 is achieved by both the positive and negative regulation of RORs and REV-ERBs. The Period (*Per*) genes are key circadian regulators in mammals that are expressed rhythmically in the SCN as well as in areas outside the SCN. The *Per2* gene is an essential component of the mammalian circadian clock and plays a primary role in the human circadian clock since the *PER2* ^*S662G*^ mutation causes the familial advanced sleep phase syndrome (Toh et al. 2001; Xu et al. 2005). It is known that genetic ablation of mPER1 and mPER2 function results in a complete loss of circadian rhythm control based on wheel-running activity in mice (Lee 2005). The circadian expression of *Per2* is regulated by the transcription factors CLOCK and BMAL1 but also via CREB (cAMP response element binding protein)-dependent transcriptional activation. Among the ATF/CREB family proteins, ATF4 binds to the CRE of the *Per2* promoter in a circadian time-dependent manner and periodically activates the transcription of the *Per2* gene (Koyanagi et al. 2011).

The circadian system is linked to various physiological processes through clock-controlled genes and the synthesis of products which control DNA synthesis, cell division, and proliferation (Gréchez-Cassiau et al. 2008; Matsuo et al. 2003; Nagoshi et al. 2004; Wood et al. 2006). Clock-related cell cycle progression has evolved to confine DNA replication to the moment of the day when the risk of exposure to environmental and endogenous DNA damaging agents (i.e., UV during the day; reactive oxygen species and other harmful metabolic side products generated during respiratory metabolism) is at its lowest level (Roenneberg and Merrow 2007; Tauber and Kyriacou 2005). DNA repair is a fundamental cellular activity that has evolved to preserve genome stability when environmental conditions or endogenous genotoxic agents endanger an organism’s health and life span. Experimental evidence has demonstrated that DNA repair is controlled by the circadian clock and that XPA, the DNA repair protein, is controlled by the circadian clock in the mouse brain, liver, and skin (Gaddameedhi et al. 2011; Kang et al. 2010). It has been found that the activity of nucleotide excision repair (NER) is highest in the afternoon/evening hours and lowest in the night/early morning hours in mice brains (Kang et al. 2009). The circadian clock regulates both DNA sensitivity to UV damage and the efficiency of NER by controlling chromatin condensation (Bee et al. 2015) as well as the repair of 8-oxoG DNA (Manzella et al. 2015). Clock components BMAL1-CLOCK, PER1, PER2, and ROR are involved in controlling the cellular response to genotoxic stress (Gaddameedhi et al. 2011; Kang et al. 2009; Miki et al. 2013).

Epidemiological studies have shown that a disruption in circadian rhythms leads to increased susceptibility to cancer in humans. Indeed, several studies have shown that rotating shift workers have an elevated risk for breast and prostate cancer (Flynn-Evans et al. 2013; Knutsson et al. 2013; Stevens 2005). The International Agency for Research on Cancer (IARC) has, in fact, classified shiftwork involving circadian disruption as probably carcinogenic to humans (Straif et al. 2007). Accumulating evidence suggests that alterations in the DNA-damage response (DDR) pathway induced by circadian rhythm dysregulation (disruption or desynchronicity) could be responsible for tumorigenesis. DDR is a complex pathway consisting in damage sensors, mediators, signal transducers, and effectors that are involved in DNA damage checkpoints and repair, apoptosis, and transcriptional reprogramming. As far as DNA damage is concerned, double-strand breaks (DSBs) represent the most dangerous lesions for genome integrity as they promote genome rearrangements that initiate carcinogenesis or apoptosis. Ataxia-telangiectasia mutated (ATM) is a member of the phosphatidylinositol 3-kinase-related kinase (PIKK) family and is the primary transducer of DSB-induced signaling. In the presence of DSBs, ATM undergoes autophosphorylation which promotes its activation to phosphorylate proteins that control signal transduction, cell cycle progression, and DNA repair. Following DNA damage, the ATM kinase phosphorylates the C′ terminus of MDM2 and the BOX-I of p53, switching MDM2 from binding the p53 protein and promoting p53 stabilization and activation (Karakostis et al. 2016). Following ATM-mediated activation, the TP53 tumor suppressor exerts its transcription regulatory activity mostly through direct binding to the regulatory sequences of its target genes. Among these, several genes belonging to the p53 pathway are under the control of the circadian clock, such as *c-MYC*, *CDKN1A* (encoding cyclin-dependent kinase inhibitor P21), and *GADD45A*. The *c-MYC* oncogene is a first-order clock-controlled gene with a role in cellular proliferation (Fu et al. 2002). *CDKN1A* is a second-order clock-controlled gene, negatively regulated by REV-ERBα, which is involved in G_1_/S and intra-S-checkpoint regulation but also in base excision repair (Gréchez-Cassiau et al. 2008).The cell cycle gene *GADD45A* , whose transcript levels are increased following stressful growth arrest conditions, is under the indirect circadian control of the essential *Per2* clock gene (Canaple et al. 2003). Although several studies have investigated the relationships between the circadian clock, cell cycle progression and apoptosis induction (Fu et al. 2002; Gery et al. 2006; Matsuo et al. 2003; Kowalska et al. 2013), the mechanisms underlying the circadian regulation of DDR pathways have not been completely elucidated.

The current study set out to investigate if cellular response to DNA damage is affected by the circadian phase at which cells are stressed. Human primary fibroblasts were thus irradiated at circadian times corresponding to the trough and the peak of the PER2 protein expression, often used as a marker of circadian time and considered a circadian readout in the context of this study. Clonogenic cell survival, DSB repair kinetics, and TP53 protein expression were analyzed as the endpoints of the DNA-damage response. The experiments were performed in non-proliferating human lung fibroblasts in order to separate the cell cycle from the circadian clock.

## Materials and methods

### Cell cultures and circadian rhythm induction

**Fig. 1:**
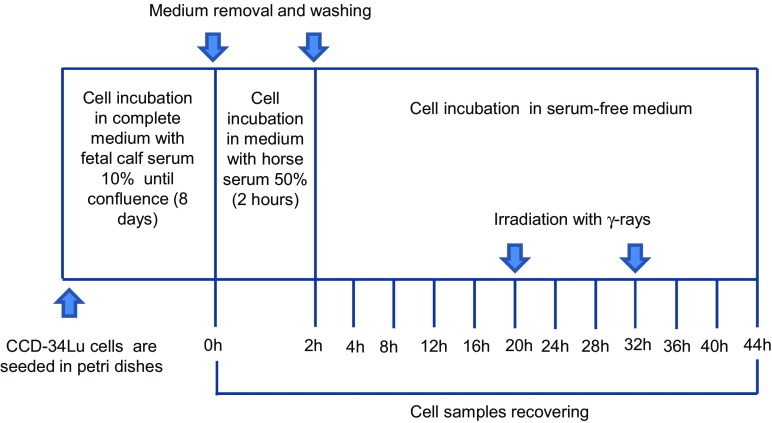
The experimental procedure to induce circadian rhythmicity in non-proliferating human fibroblasts CCD-34Lu

Normal human neonatal lung fibroblasts CCD-34Lu (ATCC N. CRL-1491™) were grown in high glucose (4.5 g/l) Dulbecco’s modified Eagle’s medium (DMEM) containing GlutaMAX (Gibco, Life Technologies), supplemented with 10% heat-inactivated fetal calf serum (FCS, Biochrom KG, Seromed), HEPES 20 mM (Sigma-Aldrich), and 1% MEM non-essential amino acids (Gibco, Life Technologies). At the time the experiments were carried out, the cells were at 13 to 16 population doublings and were actively proliferating, as confirmed by flow cytometry analysis of DNA content (Supplementary Fig. S1). Cultures of non-proliferating CCD-34Lu were obtained by seeding cells (1 × 10^6^) in 100 × 20 mm Petri dishes in a culture medium supplemented with 10% FCS until cells reached confluence (8 days); they were then shifted to a medium containing 50% horse serum (Life Technologies) and incubated for 2 h at 37 °C. At the end of the incubation period, the cells were incubated in fresh serum-free medium during the entire 44-h post-serum-shock treatment period (Fig. 1).

### Cell irradiation

Cells were irradiated at the Department of Oncological and Surgical Sciences of Padova University with a ^137^Cs source (dose rate of 2.8 Gy/min) at trough and peak PER2 protein expression. Control cells were subjected to all of the same experimental conditions as the irradiated one, except for irradiation.

### Clonogenic assay

After irradiation, the cells were harvested by trypsinization and counted by trypan blue dye exclusion. Five hundred viable CCD-34Lu cells were seeded together with feeder layer IMR90 cells (15 × 10^5^ cells/plate) in complete medium supplemented with 15% FCS, as described elsewhere (Fede et al. 2012). Four replicate dishes were prepared for each experimental point, and the cells were incubated for 12 days to allow the colonies to develop. The colonies were stained with 0.4% crystal violet before counting, and only colonies containing more than 50 cells were scored as survivors. Cell survival was calculated as the percentage of cloning efficiency (CE) of the irradiated cells over that of non-irradiated control cells.

### FACS analyses

The distribution of cells was assessed by flow cytometry analysis of cellular DNA content, as described elsewhere (Canova et al. 2005). The cells were trypsinized, counted, and seeded at low density in new dishes in completely fresh medium. Three, 24, 30, and 48 h following re-plating, the cells were recovered and analyzed after they were stained with propidium iodide (PI) (50 μg/μL) and RNase (100 μg/ml). The samples were analyzed using a BD FACSCanto™ II flow cytometer (BD Biosciences); data from 10 × 10^3^ cells/sample were collected for acquisition and cell cycle distribution analysis using CellQuest (version 3.0, BD Biosciences) and ModFit LT 3.0 software (BD Biosciences), respectively.

### Transfection with siRNAs

Confluent non-proliferating CCD-34Lu cells were transfected with 32 nM of Stealth RNAi™ siRNA anti-*PER2* (Life Technologies) or siRNA negative control (Life Technologies) using RNAiMAX (Life Technologies). The siRNA HSS113093 (Supplementary Fig. S2) resulted as the most efficient in *PER2* downregulation and was chosen for siRNA-transfection experiments. Transfections were performed in DMEM medium without antibiotics supplemented with 0.1% FBS, and after 3 days, the medium was diluted 1:1 with fresh DMEM-0.1% FBS and cells were incubated for an additional 3 days in the presence of 16 nM siRNA. CCD-34Lu cells were then transfected a second time with 32 nM of siRNA in DMEM-0.1% FBS (Franzolin et al. 2013). After 24 h, the cell medium was exchanged with DMEM containing 50% horse serum, incubated for 2 h at 37 °C, washed twice, and incubated in the previous medium containing siRNAs during the whole 44-h period after serum shock.

### Total RNA isolation and qRT-PCR

At the end of incubation periods after serum-shock synchronization, total RNA was isolated from CCD-34Lu cells using TRIzol® Reagent (Invitrogen, CA) following the manufacturer’s instructions. Total RNA quantification and RNA integrity evaluation were performed using the ND-1000 spectrophotometer (Nanodrop, Wilmington, DE) and the Agilent Bioanalyzer 2100, as described elsewhere (Girardi et al. 2012). One microgram of total RNA was retro-transcribed with ImProm-II Reverse Transcription System (Promega). Quantitative real-time PCR (qRT-PCR) was performed with the GoTaqqPCR Master Mix (Promega) and gene-specific primers for *PER2*, *BMAL1*, *TP53*, *CDKN1A*, *GADD45A*, and *c-MYC* genes and for *GAPDH* as reference. qRT-PCR reactions were always performed in quadruplicates. The gene expression levels were calculated using the comparative delta threshold cycle (CT) method (2^−∆CT^) implemented in the 7500 Real-Time PCR System software (Livak and Schmittgen 2001).

### Immunofluorescence staining

Immunofluorescence detection of γ-H2AX and 53BP1 foci was used to detect DNA-damage induction and repair. The cells (0.4 × 10^6^) were seeded on Petri dishes (60 × 15 mm) containing coverslips and were treated for circadian clock synchronization. They were then irradiated at trough and peak PER2 expression and cultured at 37 °C in fresh serum-free medium for different repair times (0.5, 2, 6, and 24 h). At each time point, the non-irradiated and irradiated cells were rinsed once with cold PBS and fixed with a 4% solution of formaldehyde (Sigma-Aldrich) at 37 °C for 15 min. The cells were washed three times for 5 min in PBS, permeabilized in 0.2% Triton X-100–PBS and blocked in 10% goat serum–PBS for 1 h at room temperature. The cells were then incubated for 2 h at room temperature with primary antibodies anti-γ-H2AX (Ser139) Clone JBW301 (Millipore, lot. no. DAM1493341, 1:100, or Abcam, ab11174, 1:100) or anti-53BP1 (Bethyl Laboratories, lot. no. A300-273A-4, 1:100) and then washed three times in PBS and once in PBS + 0.1% Triton X-100. The cells were then incubated at room temperature for 1 h with secondary antibodies Alexa Fluor® 488 goat anti-mouse and Alexa Fluor® 594 donkey anti-rabbit (Life Technologies, 1:350) and washed, as described elsewhere. Cells were washed and counterstained with 2 μg/ml DAPI (4′,6-diamidino-2-phenylindole) in antifade solution (Vectashield, Vector Laboratories), and cover glass slips were mounted. Images of γ-H2AX and 53BP1 foci were captured using a Leica TCS SP5 confocal microscope (Leica Microsystems) with a 63× oil immersion objective. All the images were acquired under the same conditions of laser intensity, PMC voltage, pinhole aperture, and optical slice (0.5 μm) and processed by Adobe Photoshop 8.0 software (Adobe). For each experimental point, foci were scored by eye from 300–500 nuclei.

### Subcellular fractionation and SDS PAGE

Cellular extracts were prepared from serum-synchronized cells 0, 5, and 24 h after irradiation and in non-irradiated cells. Nuclear extraction of proteins was performed using the CelLytic™ NuCLEAR™ Extraction Kit (Sigma-Aldrich), following the manufacturer’s instructions. Briefly, cells (2.5 × 10^6^) were suspended in Lysis Buffer 1×, containing DTT and protease inhibitors, incubated on ice 15 min allowing cells to swell. Then a 10% IGEPAL CA-630 solution to a final concentration of 0.6% was added to the swollen cells. Cells were vortexed and centrifuged at 10.000×*g* for 30 s, and the cytosolic fraction was recovered. The nuclei pellet was resuspended in Extraction Buffer containing DTT and protease inhibitors, vortexed for 30 min, and centrifuged at 21.000×*g* for 5 min, and supernatant containing the nuclear fraction was collected. Ten micrograms of nuclear fraction were separated on 10% SDS–polyacrylamide gels and transferred to nitrocellulose membranes (Hybond-C Extra; Amersham, GE Healthcare). Membranes were then probed with primary antibodies anti-PER2 (H-90, sc-25363, Santa Cruz, 1:200), anti-p53 (Ser20) (1C12, Cell Signaling, 1:100), anti-phospho-p53 (Ser15) (16G8, Cell Signaling, 1:100), and anti-GAPDH (Millipore, 1:5000) and then incubated with Amersham ECL horseradish peroxidase-conjugated secondary antibodies (GE Healthcare, 1:40.000). The resulting immunoreactive bands were detected using enhanced chemiluminescent HRP substrate (Millipore). The bands’ intensities were quantified with ImageJ software and normalized utilizing GAPDH.

### Statistical analysis

All data are expressed as the means ± standard deviation (SD) of at least three independent replicate experiments. Student’s *t* test was used to analyze differences between two groups of clonogenic assay. Comparisons between multiple groups of ionizing radiation-induced foci were made using two-way analysis of variance (ANOVA) and Tukey’s post hoc test. Values of *p* < 0.1 were considered to be statistically significant.

## Results

### Induction of circadian rhythm in human fibroblast cultures

Unlike the *Drosophila* and zebrafish model organisms in which the peripheral clock can be entrained directly by light (Ceriani et al. 1999; Whitmore et al. 2000), one or more blood-borne factors are required in mammals to stimulate signal transduction pathways that influence the molecular oscillators in peripheral cells. In vitro, brief treatment of cultured rat1 fibroblasts with various compounds (serum, forskolin, cAMP or dexamethasone, etc.) induces rhythmic expression of clock genes (*Per1*, *Per2*, *Cry1*) and the circadian transcription factors (REV-ERBα, DBP, and TEF) for two to three consecutive daily oscillations, with an average period of 22.5 h (Allen et al. 2001; Balsalobre et al. 1998). Circadian rhythm is successfully induced by serum shock and cAMP analogs in cultured human mesenchymal stem cells (Huang et al. 2009).

**Fig. 2:**
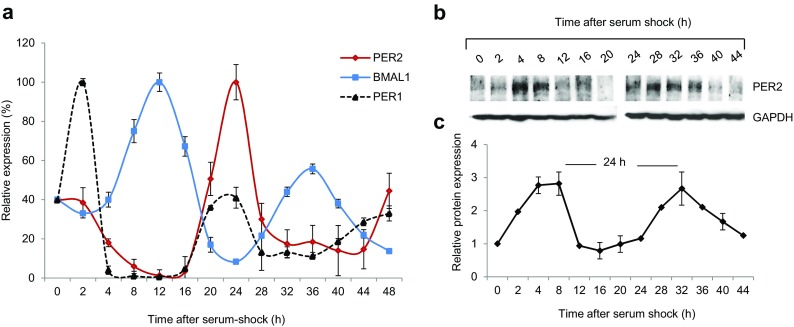
Induction of circadian rhythm in serum-shocked human CCD-34Lu fibroblasts. **a** Expression of *PER1*, *PER2*, and *BMAL1* transcripts obtained using qRT-PCR analysis at the indicated time points beginning at serum shock (0 h). The values are normalized with *GAPDH* mRNA as an internal control and plotted as percentages (means ± SD). **b** Representative western blot of PER2 protein at the same time points after serum shock and its quantification expressed in the fold-change (FC) relative to GAPDH protein as loading control (**c**)

**Fig. 3:**
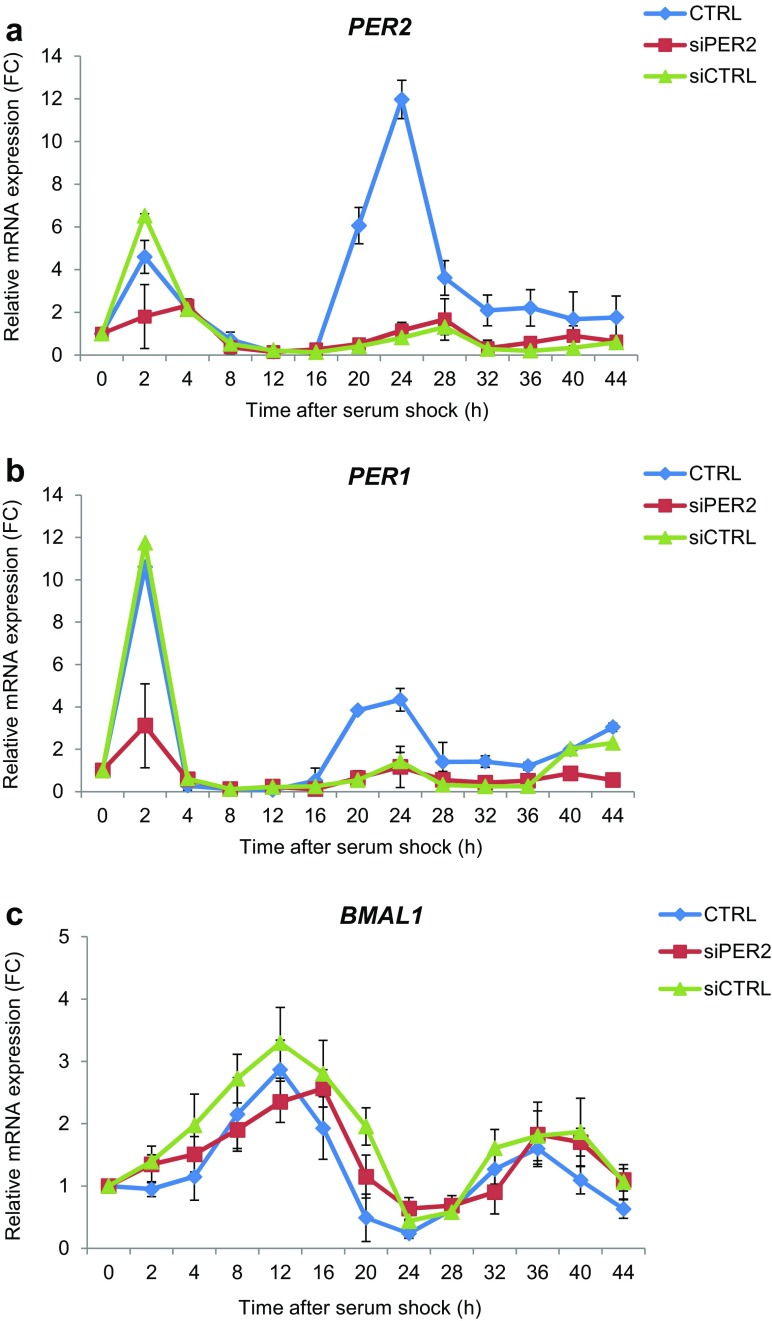
Role of *PER2* in the circadian phase of serum-shock synchronized human fibroblasts CCD-34Lu. Relative expression measured by qRT-PCR analysis of *PER2*(**a**), *PER1* (**b**), and *BMAL1* (**c**) transcripts in cells transfected with si*PER2* and si*CTRL* and for comparison in untransfected control cells (CTRL) analyzed at the indicated time points beginning at serum shock (0 h).The values (means ± SD) are normalized with *GAPDH* mRNA as an internal control and plotted as fold-change

The experiments performed in this study were carried out in non-proliferating CCD-34Lu fibroblasts in order to maintain the cells and circadian cycles separated while they were being exposed to genotoxic stress. The cells were grown in normal culture conditions until they reached confluence (G_1_ phase), which was confirmed by flow cytometric analyses (Supplementary Fig. S1). They were then incubated for 2 h in fresh medium containing horse serum (50%). At the end of serum shock, a serum-free medium was used and the cells were incubated for 44 h (Supplementary Fig. S2). To verify the rhythmic expression of clock genes, we analyzed the expression of *PER1*, *PER2*, and *BMAL1* mRNAs at the beginning and at the end of serum shock and every 4 h for a 44-h interval. Both *PER1* and *PER2* transcripts confirmed a circadian expression within approximately a 24-h period, with a trough at 12 h and a peak at 24 h after serum shock, although *PER2* induction was more evident (Fig. 2a). *BMAL1* transcript was, as expected, in anti-phase with *PER1* and *PER2*, with the trough at 24 h after serum shock and two peaks at 12 and 36 h, respectively, after serum shock. We then analyzed the expression level of PER2, the key mammalian circadian clock protein, which showed two peaks of induction, at 4–8 and 28–36 h after serum shock, and a trough one at 12–20 h after serum shock (Fig. 2b, c), confirming a circadian oscillation of about 24 h. To evaluate the role of *PER2* in the circadian phase of our system, we performed experiments of *PER2 s*ilencing in CCD-34Lu cells subjected to serum shock. We first analyzed the expression of *PER2* transcript in cells transfected with si*PER2* or si*CTRL* to verify the effect of silencing throughout the 0–44-h period after serum shock, and then we analyzed the expression of *PER1* and *BMAL1* transcripts by qRT-PCR (Fig. 3). Our results show that the *PER1* peak expression (at 2 and 24 h after serum shock) was reduced after *PER2* knockdown, whereas *BMAL1* expression level maintained on the whole a circadian rhythmicity, within approximately a 24-h period even though its peak expression was 4 h shifted in si*PER2* cells (16 h instead of 12 h after serum shock).

### Clonogenic survival in cells irradiated at trough and peak PER2 protein expression

**Fig. 4:**
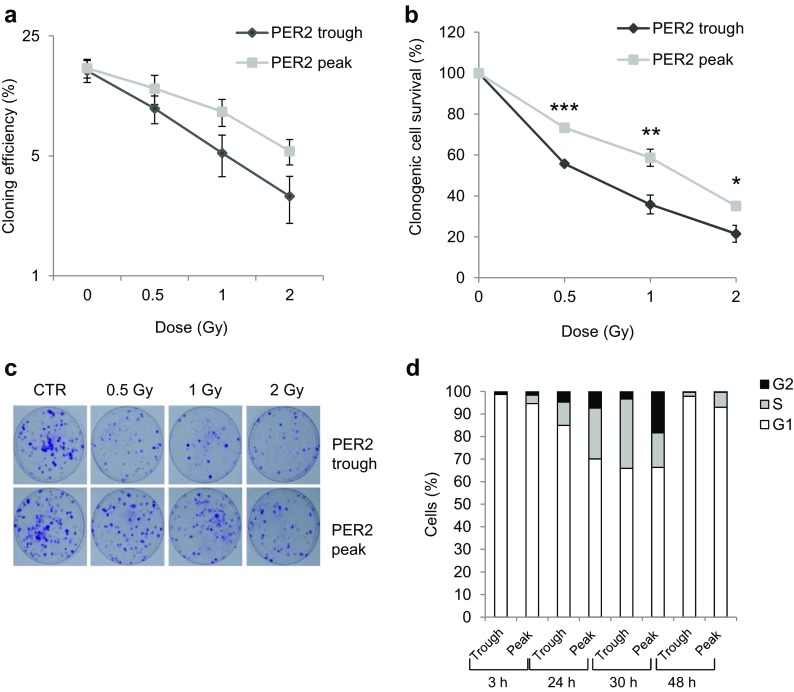
Clonogenic cell survival of serum-shocked γ-irradiated human CCD-34Lu fibroblasts. **a** The cloning efficiency (CE) of serum-shocked non-irradiated (0 Gy) cells and of cells irradiated with 0.5-1-2 Gy at the trough and the peak of PER2 protein expression, as a marker of the circadian clock. **b** Cell survival rate was calculated as the percentage of CE of irradiated cells with respect to that of non-irradiated control cells treated with serum shock and analyzed at the same circadian times. Data refer to means ± SD of independent experiments (***p < 0.001, **p < 0.01, *p < 0.05; *t* test). **c** Colony formation in representative dishes of non-irradiated (*CTR*) and irradiated cells recovered at the trough and the peak of PER2 expression. **d** Cell cycle analyses in cells recovered at the trough and the peak of PER2 protein expression, re-plated at low density, and analyzed 3, 24, 30, and 48 h later. Data were collected from 10.000 cells/sample using a BD FACSCanto™ II flow cytometer

We investigated the role of PER2 expression level in regulating the cellular response to DNA damage by first analyzing cell survival in relation to the circadian phase during which cells were exposed to ionizing radiation (IR). To this aim, we selected circadian times corresponding to the trough and the peak of PER2 protein expression, as a marker of circadian phase, in order to administer genotoxic stress at those times. In accordance with previous results, PER2 protein was maximally induced at 28–36 h after serum shock and minimally expressed between 12 and 20 h after serum shock (Bee et al. 2015). We thus chose two circadian times separated by a 12-h interval (i.e., 20 and 32 h) as respectively the trough and the peak of PER2 protein expression. We assessed cell survival after irradiation, performed at the trough and the peak of PER2 protein expression, by determining the cloning efficiency (CE) of irradiated and non-irradiated control cells (CTRs) that were subjected to the same treatments except irradiation. As previously reported, cells irradiated at the peak of PER2 expression displayed cloning efficiency values that were typical of this cell line (Bee et al. 2013). The cells irradiated at trough PER2 expression were less able in general to form colonies with respect to the cells irradiated at its peak (Fig. 4a) and showed decreased cell survival at all doses (Fig. 4b; *p* < 0.001 for 0.5 Gy; *p* < 0.01 for 1Gy; *p* < 0.05 for 2 Gy, *t* test). As the non-irradiated control cells showed similar CE values at both circadian times, this would seem to indicate that their ability to form colonies did not change as a function of the time the cells were kept in serum-free medium. Cell cycle analysis was also performed on the non-irradiated cells recovered at PER2 protein trough and peak, re-plated at low density (25 × 10^4^ cells/cm^2^) in fresh medium supplemented with serum and analyzed 3, 24, 30, and 48 h later. Re-plating in fresh complete medium stimulated cell growth in both cell groups, and despite the fact that 24 h after re-plating the S-G_2_ fraction was higher in the cells harvested at the peak than at the trough of PER2 expression, 30 h after re-plating, the S-G_2_ fraction was similar in the two cell groups (∼34%, Fig. 4d). Cell growth analysis was performed also in serum-shocked CCD-34Lu cells irradiated with 1 and 5 Gy at the peak and trough of PER2, harvested, re-plated at low density, and recovered at 24, 30, and 48 h after irradiation (supplementary Fig. 3). At 24 h after re-plating, the S-G_2_ fraction in 1-Gy-irradiated cells was similar in both cell groups (∼25%), whereas at 30 h after re-plating, the S-G_2_ fraction was higher in cells irradiated at the trough than at the peak of PER2 expression (40 vs. 17%, respectively); at 48 h after re-plating, most of the cells were G_1_-arrested in both cell groups but the S-G_2_ fraction was still higher in cells irradiated at PER2 trough (11 vs. 3%). These findings suggest that the circadian effect related to PER2 expression manifests on the proliferation ability of both isolating cells (i.e., during clonogenic assay) and cells growing in culture. In 5-Gy-irradiated cells, no evident differences of cell growth emerged from the two groups since most of the cells was in G_1_-phase starting from 24 h after re-plating in accordance with previous data (Bee et al. 2013).

### Analysis of DSB repair kinetics in cells irradiated at trough and peak PER2 protein expression

**Figure 5:**
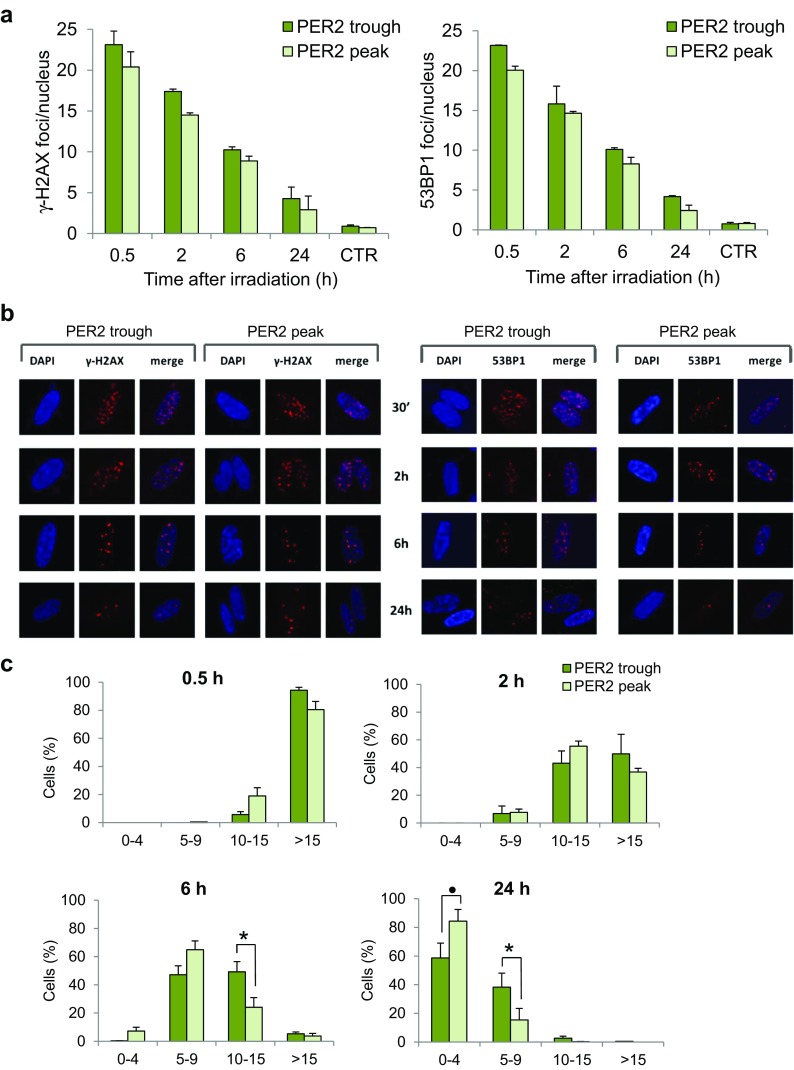
The kinetics of ionizing radiation-induced foci (IRIF) in serum-shocked γ-irradiated human CCD-34Lu fibroblasts. The mean number of γ-H2AX foci and 53BP1 foci (**a**) after irradiation administered at the trough and the peak of PER2 protein expression. **b** Representative immunofluorescence of γ-H2AX and 53BP1 foci in nuclei stained with DAPI at 0.5, 2, 6, and 24 h after irradiation. **c** Cells were categorized as having 0–4, 5–9, 10–15, and >15 of γ-H2AX and 53BP1 foci/nucleus. Data are means ± SD from independent experiments, each with at least 200 nuclei/time points, carried out in cells irradiated at the trough and the peak of PER2 protein expression (^*•*^ *p* < 0.1, *p < 0.05; ANOVA test)

In response to DSBs, the phosphorylation of histone H2AX (γ-H2AX) is one of the earliest ATM-dependent responses to IR, which is necessary for the accumulation of numerous essential proteins in irradiation-induced foci (IRIF) and playing a critical role in recruiting DNA-damage signaling and repair proteins at DSB sites (Celeste et al. 2003). Among these, the tumor suppressor p53-binding protein 1 (53BP1) becomes hyperphosphorylated after irradiation and colocalizes with phosphorylated H2AX in megabase regions surrounding the sites of DNA strand breaks (Rappold et al. 2001; Schultz et al. 2000), and the time course of 53BP1 foci formation/disappearance is similar to that of γ-H2AX foci. Since the number of foci is correlated with the number of DNA DSBs (Sharma et al. 2012), we monitored the kinetics of DSBs by in situ immunofluorescence of IRIF of γ-H2AX and 53BP1 proteins. The cells were irradiated at the trough and peak PER2 protein expression and then fixed at different time points (0.5, 2, 6, 24 h) after irradiation. The quantification of foci per nucleus showed that DSB resolution proceeded with similar kinetics in both cell groups, although cells irradiated when PER2 protein was minimally expressed generally showed ∼2–4 more IRIF/nucleus than cells irradiated at peak PER2 expression at all time points after IR (Fig. 5a). By categorizing the cells as having 0–4, 5–9, 10–15, and >15 foci/nucleus, it nevertheless emerged that a significant fraction of the cells irradiated at the trough PER2 expression retained more IRIF/nucleus with respect to the cells irradiated at its peak. In particular, for time 6 and 24 h, the resulting interactions were significant with two-way ANOVA test (respectively *p* = 8.17e−10 and *p* = 1.61e−10). Then, using Tukey HSD test for ANOVA, we found a significant difference for the categorization 10–15 at 6 h (*p* = 0.024) and for the categorizations 5–9 (*p* = 0.025) and 0–4 at 24 h after irradiation (*p* = 0.077) (respectively at 6 h and at 24 h after IR, Fig. 5c).

### Analysis of TP53 expression in serum-shocked CCD-34Lu cells

**Fig. 6:**
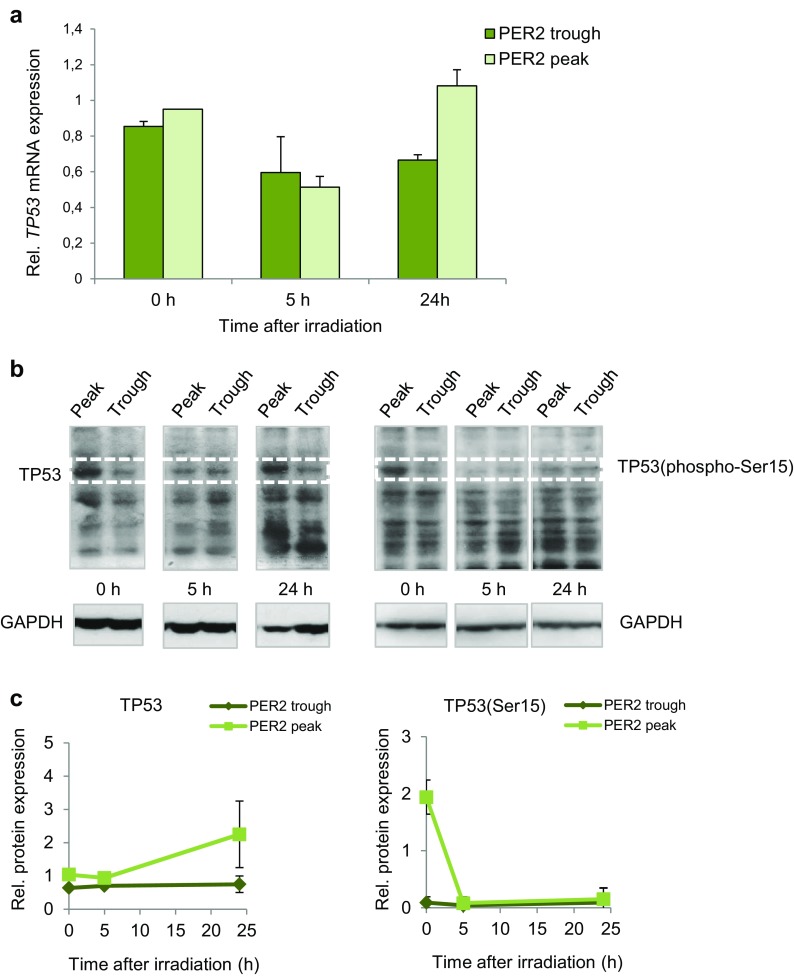
Analysis of TP53 expression in serum-shocked CCD-34Lu cells irradiated with γ-rays at circadian times corresponding to the peak and the trough of PER2 protein expression. **a** The relative expression of the *TP53* transcript was measured at 0, 5, and 24 h after irradiation with γ rays (5 Gy); data are means ± SD expressed as fold-change of irradiated vs. non-irradiated cells. **b** TP53 protein accumulation in nuclear extracts of cells irradiated with 5 Gy at the trough and the peak of PER2 protein expression and harvested at 0, 5, and 24 h after irradiation. Representative western blot of TP53 and phosphorylated-TP53(Ser15) proteins and GAPDH as loading control. **c** Relative expression of TP53 and phosphorylated-TP53(Ser15) proteins; the bands’ intensities were quantified with ImageJ software and normalized using GAPDH

Since the *TP53* gene is pivotal in the DDR process, we analyzed the expression of its transcript in serum-shocked cells irradiated with 5 Gy at the peak and the trough of PER2 protein expression and analyzed them 0, 5, and 24 h after irradiation (Fig. 6a). The expression level of *TP53* mRNA was downregulated at 5 h after IR in both cell groups, and at 24 h after, IR returned to control value (∼1) in the cells that were irradiated at the peak of PER2 protein expression. To verify whether TP53 protein was affected in the cells irradiated at different circadian times, we used western blot to assess the levels of nuclear TP53 protein in the cells irradiated at the peak and the trough of PER2 expression and harvested very early (0 h), 5, and 24 h after irradiation (Fig. 6b, c). The results showed that TP53 protein accumulated in the cells irradiated at the peak of PER2 protein expression, in particular at 0 and 24 h after IR, whereas it was poorly detectable in the cells irradiated at the trough of PER2 expression. To further analyze TP53 behavior, we also assessed the nuclear phosphorylated TP53(Ser15) levels at the same times after irradiation. Phospho-p53 was detectable very early after irradiation in the cells irradiated at the peak but not at the trough PER2 expression and very poorly detectable at later time points after irradiation in either cell group.

### Expression analysis of p53-related genes in cells irradiated at trough and peak PER2 expression

**Fig. 7:**
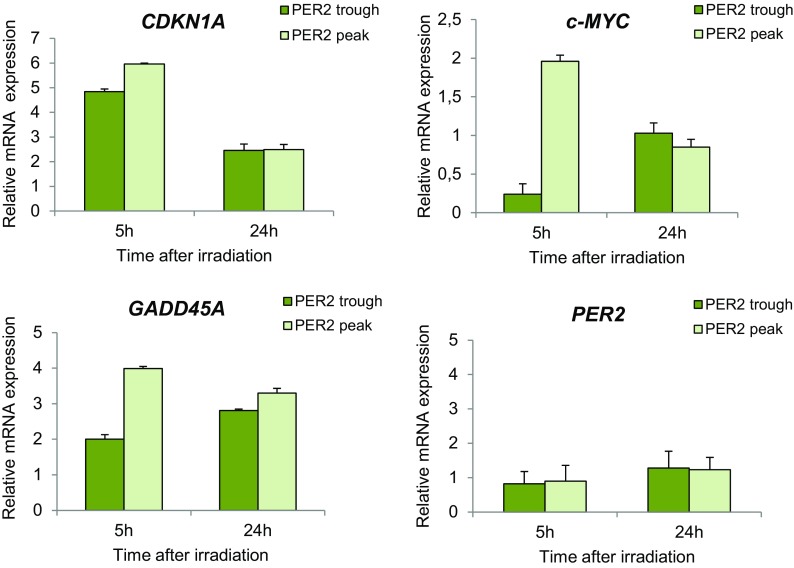
Expression level of p53-related genes in serum-shocked human fibroblasts CCD-34Lu irradiated with γ rays. The relative expression of *CDKN1A* (p21), *GADD45A*, and *c-MYC* transcripts was measured at 5 and 24 h after irradiation (5 Gy)performed at the trough and the peak of PER2 protein expression. The relative expression of *PER2* transcript is reported at both irradiation times. Values (mean ± SD) are expressed as fold-change of irradiated vs. non-irradiated control cells of each group

We evaluated whether the activity of TP53 as transcriptional regulator was affected in relation to PER2 expression by analyzing the expression level of the p53-related genes *CDKN1A* (p21), *c-MYC*, and *GADD45A* in CCD-34Lu cells irradiated at trough and peak PER2 expression. At 5 h after irradiation, the transcription of *CDKN1A* was five- to sixfold induced in both cell groups and then decreased at 24 h after irradiation, being approximately twofold over the control in both cell groups (Fig. 7). *c-MYC* showed a twofold induction at 5 h after irradiation in cells irradiated at peak but not at trough of PER2; at 24 h after irradiation, its level was similar to that of control in both cell groups. *GADD45A* was more induced in cells irradiated at PER2 peak than trough (fourfold vs. twofold, respectively) at 5 h after irradiation; at 24 h after irradiation, its expression level decreased and was similar in both groups (approximately threefold over control). The expression level of *PER2* gene was unaffected in the two cell groups at both time points after irradiation.

## Discussion

The disruption of circadian rhythmicity or irregular circadian cycles, such as those experienced by night-shift workers in humans or caused by constant exposure to light in rodents, have been associated with higher cancer risk (Anderson et al. 2000; Hansen 2006; Megdal et al. 2005; Sigurdardottir et al. 2012; van den Heiligenberg et al. 1999). *Per* genes are mutated or deleted in many tumor tissues, and altered *PER2* expression is common in human breast cancer (Chen et al. 2005; Winter et al. 2007) and in patients with prostate cancer (Kiss and Ghosh 2016). The *PER2* ^*S662G*^ mutation has been found to be responsible for abnormal DNA-damage response (DDR) and increased cancer risk (Gu et al. 2012). Mice deficient in the *mPer2* gene were found to be cancer-prone. These mice showed, in fact, a marked increase in tumor development and reduced apoptosis after gamma-irradiation (Fu et al. 2002). Alterations in the expression of critical clock genes have been reported in both breast (Chen et al. 2005; Yang et al. 2009) and gastric cancer (Hu et al. 2014). It has recently become apparent that DNA repair, transcriptional reprogramming, and apoptosis exhibit daily fluctuations, suggesting that the time of exposure to genotoxic stress could affect cell response.

The current study focused on the cellular response to DNA damage that was induced by ionizing radiation (IR) in normal human CCD-34Lu fibroblasts to clarify the relationships between the circadian rhythmicity and the DNA-damage response pathway. Since cell proliferation depends on extracellular mitogen-activated signals, it is temporally controlled by the circadian clock and these interactions may have both physiological and pathological implications. For these reasons, we analyzed normal non-proliferating fibroblasts, in which circadian rhythm was induced by applying a 2-h pulse of horse serum, a method that has been used to synchronize clock gene expression in mammalian cells (Balsalobre et al. 1998; Huang et al. 2009). Here, it was successfully applied to non-proliferating CCD-34Lu cells, as demonstrated by the time course of *PER1*, *PER2*, and *BMAL1* gene expression and PER2 protein (Fig. 2). The results concord with recent data obtained in quiescent human skin fibroblasts showing the same circadian times of PER2 maximum and minimum expression after dexamethasone pulse (Bee et al. 2015). PER2 is an important component of the circadian system, and its role has been clarified to some extent by several previous studies. Clock gene *Per2* is robustly and rhythmically expressed in almost all mammalian tissues (Albrecht et al. 2007), and under physiological conditions, *PER2* oscillates rhythmically showing a peak and a trough that are in anti-phase with the other important clock gene *BMAL1*. Circadian oscillators are robust also in in vitro cultured fibroblasts which harbor self-sustained and cell-autonomous circadian clocks similar to those operative in SCN neurons (Nagoshi et al. 2004). In our cell system, the knockdown of the *PER2* gene affected the normal rhythmic expression of the *PER1* gene, whose level was downregulated, whereas we found no marked alterations in the mRNA expression of *BMAL1* in *PER2*-silenced cells, suggesting that *BMAL1* is not subject to PER2 regulation at the transcriptional level in CCD-34Lu cells (Fig. 3). However, since our data are only concentrated on the transcriptional level, further studies on the translational and post-translational levels are needed to investigate the role of PER2 in the clock gene network of human normal fibroblasts.

Similar to those that normally occur during medical or accidental radiation exposure, it has been shown that the γ-ray doses utilized during the current study elicit a DDR in CCD-34Lu cells (Bee et al. 2013). At the time of exposure to DNA damage, the CCD-34Lu cells were non-proliferating and stimulated after IR to enter the cell cycle to originate colonies. Study results demonstrating that cells irradiated at the trough and not at the peak of PER2 were affected with regard to their clonogenic capacity concord with reports showing increased sensitivity to γ radiation in *Per2* mutant mice (Fu et al. 2002) and decreased radio sensitivity in proliferating NIH 3T3 fibroblasts overexpressing *mPer2* (Chang et al. 2009). But data regarding clonogenic survival in clock-altered cells exposed to genotoxic stress showed quite a different picture. Overexpression of clock gene *Per1* in human cancer cell lines led to significant growth reduction following ionizing radiation (Gery et al. 2006), whereas mouse cell lines mutated in clock genes were indistinguishable from wild-type ones in their response to ionizing radiation and other DNA damaging agents (Gaddameedhi et al. 2012). These discrepancies could be due to the interconnectivity between the circadian clock and the cell cycle as well as to a strict connection between the cell cycle and the malignant phenotype. In parallel experiments carried out in cycling clock-synchronized CCD-34Lu cells, we did not evidence indeed differences in clonogenic survival between cells irradiated at the peak than at the trough of PER2 expression (data not shown).

In addition to cell survival, even DSB repair was affected in non-proliferating CCD-34Lu irradiated at the trough of PER2 protein expression; indeed, a higher number of foci were retained in the nuclei of cells irradiated at the trough with respect to the peak of PER2, indicating a delay in DSB resolution (Fig. 5). Our results are in accordance with recent evidence showing that mouse splenocytes exhibit significant differences in IR-induced DNA damage/repair response during the 24-h light-dark cycle, with faster and more efficient repair activities during the light phase of the day (Palombo et al. 2015) when the Per2 gene expression and PER2 protein levels are higher with respect to nighttime values (Zhao et al. 2015).

We also analyzed TP53 expression levels after irradiation to determine if cells with high and low endogenous levels of PER2 protein respond differently to DNA damage. In normal unstressed cells, the TP53 protein is maintained at low steady-state levels by a turnover that is predominantly regulated by MDM2-mediated ubiquitination and degradation. TP53 is stabilized in response to DNA damage because MDM2’s ability to inhibit p53-dependent transactivation is impaired (Shieh et al. 1997), meaning that it is rapidly accumulated. The circadian rhythmicity of the *TP53* gene has been observed in various tissues (Soták et al. 2014), while the expression of TP53 protein has been observed to be rhythmic in human oral mucosa (Bjarnason et al. 1999) and in malignant cells although mRNA TP53 levels did not vary (Horiguchi et al. 2013). In the current study, the *TP53* gene expression did not show a robust circadian oscillation in non-irradiated cells during the 44-h time course after serum shock (supplementary Fig. 4) nor did it show a strong induction after irradiation (Fig. 6a). Previous studies established a connection between the circadian regulatory system and the DNA-damage response mechanism at the level of Per2-p53 interaction in circadian synchronized human colorectal carcinoma cells (Gotoh et al. 2014, 2015). Very recently, Gotoh et al. (2016) proposed a unifying model for Per2-mediated regulation of p53 translocation and signaling. Under physiological conditions, the Per2:p53 interaction represents a mode of controlling the endogenous level of p53, and in cells exposed to genotoxic stimuli, such interaction is functionally relevant for modulating p53-mediated transcriptional response. Both processes are related and mediated by events that control the translocation and availability of p53 via Per2 in each subcellular compartment over the course of the circadian cycle. In our experiments, the TP53 protein was detectable after IR in the nuclei of the cells irradiated at peak PER2 expression but it was slightly detectable in cells irradiated at the trough one (Fig. 6b). To learn more about the role of PER2 expression in TP53 activation, we analyzed phosphorylated TP53(Ser15), since phosphorylation in response to DNA damage is correlated both with the accumulation of total TP53 protein and with TP53’s ability to transactivate downstream target genes in wild-type cells (Siliciano et al. 1997). Increased levels of phospho-P53 were detected very early after irradiation in the cells irradiated at the peak but not at the trough PER2 expression (Fig. 6b). At other times after irradiation, phosphorylated TP53 was poorly detectable in all the samples, in accordance with a peak of induction at 3 h after irradiation followed by degradation UBE4B-mediated of phospho-p53(S15) (Du et al. 2016). Accordingly, circadian-regulated p53-related genes *CDKN1A*, *GADD45A*, and *c-MYC* were more induced at 5 h after irradiation in cells irradiated at peak than trough PER2 expression(Fig. 7). *CDKN1A* and *GADD45A* are known to be radioresponsive genes whose expression level markedly increases following irradiation in human lymphocytes (Amundson et al. 2003; Mognato and Celotti 2005; Badie et al. 2008; Girardi et al. 2012). *c-MYC*, which is not classified as a radioresponsive gene, is nevertheless induced in human G_0_ lymphocytes following γ-irradiation (Girardi et al. 2012). On the basis of these findings, we speculate that DNA-damage response in γ-irradiated CCD-34Lu cells could depend on the cross-talk between PER2 function and TP53 protein which is relevant for modulating P53-mediated transcriptional response under stress conditions.

## Conclusions

The present study aimed to analyze the DNA-damage response to ionizing radiation in primary human lung fibroblasts when endogenous PER2 level was at its peak or trough of expression, simulating, as far as possible, conditions similar to those occurring under physiological conditions of circadian rythmicity. Taken together, our results show that clonogenic cell survival, DSB repair, and TP53 activation were affected in irradiated cells with low endogenous PER2 protein levels. Although further studies are warranted to elucidate the mechanism/s interconnecting the circadian clock and the DDR pathway, the current findings have provided more information about the DNA-damage response of human normal non-proliferating fibroblasts, reflecting the physiological condition of most cells in the adult organism. The results must nevertheless be interpreted as cell-based in in vitro systems in which the circadian component is limited in animal models.

## Electronic supplementary material

Supplementary Figure 1 Cell cycle analysis in proliferating (**a**) and non-proliferating (**b**) CCD-34Lu cells. (PDF 38 kb)

Supplementary Figure 2 Analysis of *PER2* expression by qRT-PCR in human fibroblasts CCD-34Lu transfected with siRNAs. **a** Relative expression of *PER2* transcript in cells transfected with 32 nM of three different Stealth RNAi™ siRNAs delivered alone or in combination, or siCTRL (siRNA with a nonsense/scrambled sequence) using Lipofectamine RNAiMAX. The values (means ± SD) are normalized with *GAPDH* mRNA as an internal control and plotted as fold-change. **b** Sequence of three different Stealth RNAi™ siRNAs (PER2HSS113092, PER2HSS113093, PER2HSS189627) tested to silence *PER2* gene in CCD-34Lu cells. **c** Mean Ct (threshold cycle) in CCD-34Lu cells transfected with si*PER2*n.3 or siCTRL. (PDF 19 kb)

Supplementary Figure 3 Cell cycle analyses in CCD-34Lu cells irradiated with γ-rays at the trough and the peak of PER2 protein expression. Cells were recovered after irradiation with 1 and 5 Gy, re-plated at low density (25 × 10^4^ cells/cm^2^), and analyzed 24, 30, and 48 h later. Data were collected from 10.000 cells/sample using a BD FACSCanto™ II flow cytometer. (PDF 5 kb)

Supplementary Figure 4 Analysis of *TP53* gene expression in serum-shocked synchronized human fibroblasts CCD-34Lu. Expression of *TP53* transcript obtained using qRT-PCR analysis at the indicated time points beginning at serum shock (0 h). The values are normalized with *GAPDH* mRNA as an internal control and plotted as fold-change (means ± SD). (PDF 14 kb)

## Abbreviations

DDR: DNA-damage response
DSB: Double-strand break
IR: Ionizing radiation
IRIF: Ionizing radiation-induced foci
