# Supplementary figures and images for: Analysis of DNA-damage response to ionizing radiation in serum-shock synchronized human fibroblasts
Source: Cell Biol Toxicol. 2017 May 3;33(4):373–88. doi: 10.1007/s10565-017-9394-9 (PMC5493713; doi:10.1007/s10565-017-9394-9)

**a**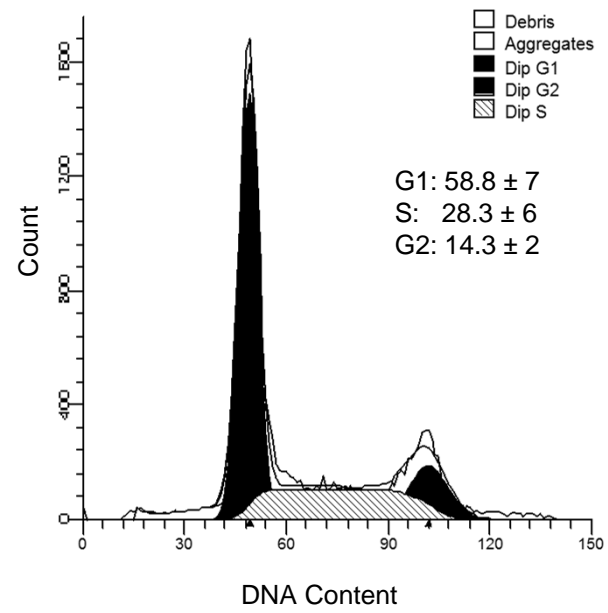**b**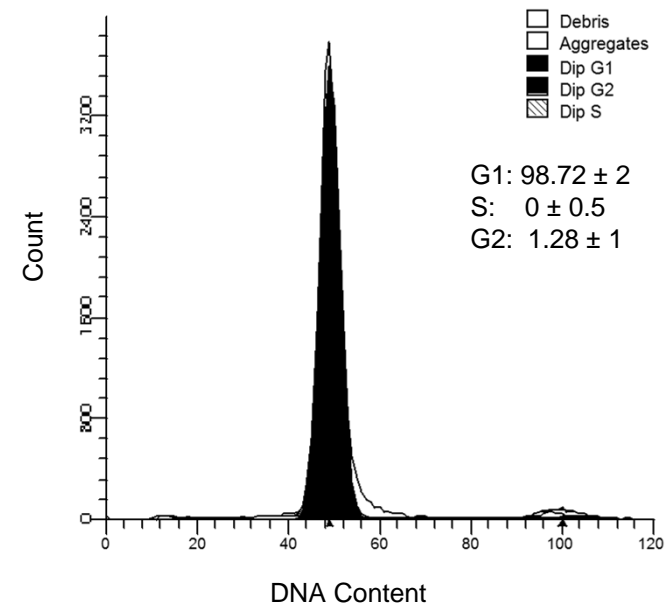

Supplement: Supplementary file 1 — Cell cycle analysis in proliferating (a) and non-proliferating (b) CCD-34Lu cells. (PDF 38 kb) [file 10565_2017_9394_MOESM1_ESM.pdf]

## 1 Gy

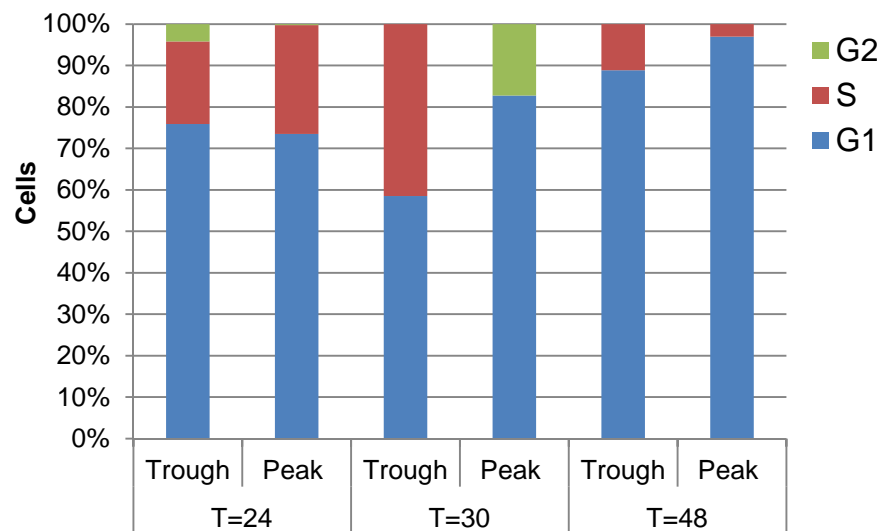

## 5 Gy

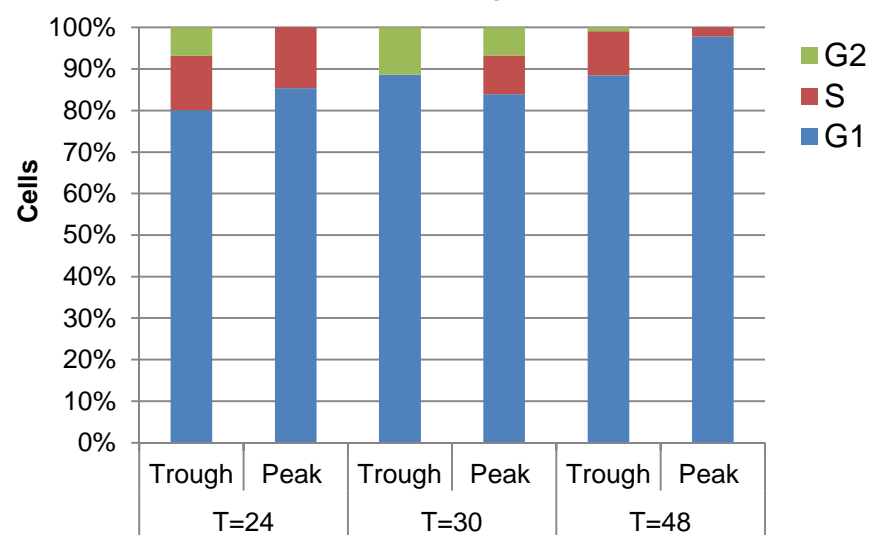

Supplement: Supplementary file 3 — Cell cycle analyses in CCD-34Lu cells irradiated with γ-rays at the trough and the peak of PER2 protein expression. Cells were recovered after irradiation with 1 and 5 Gy, re-plated at low density (25 × 104 cells/cm2), and analyzed 24, 30, and 48 h later. Data were collected from 10.000 cells/sample using a BD FACSCanto™ II flow cytometer. (PDF 5 kb) [file 10565_2017_9394_MOESM3_ESM.pdf]

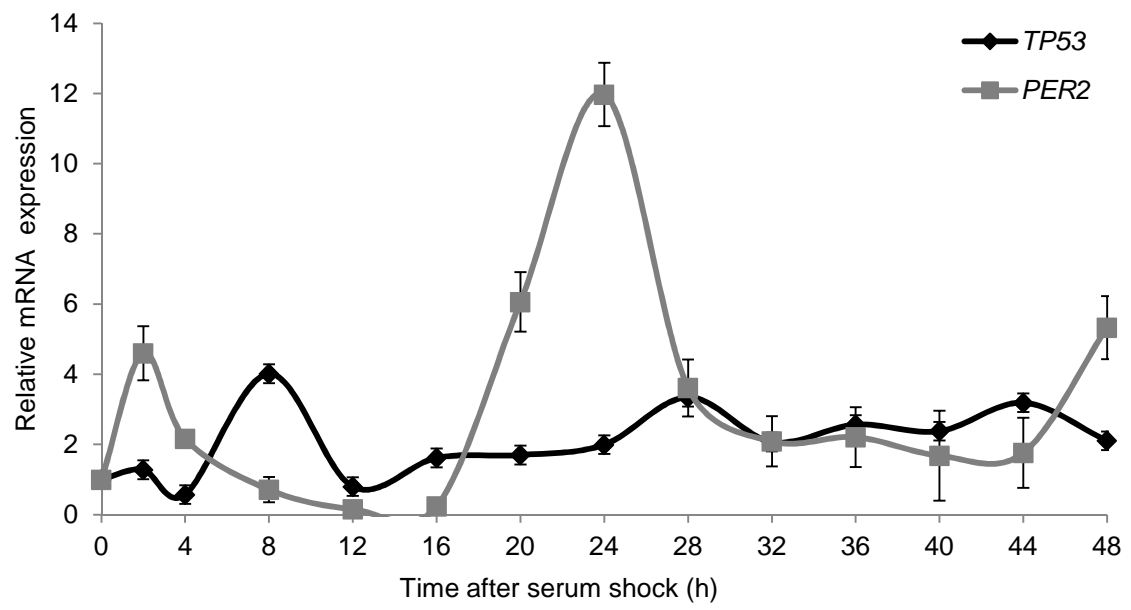

Supplement: Supplementary file 4 — Analysis of TP53 gene expression in serum-shocked synchronized human fibroblasts CCD-34Lu. Expression of TP53 transcript obtained using qRT-PCR analysis at the indicated time points beginning at serum shock (0 h). The values are normalized with GAPDH mRNA as an internal control and plotted as fold-change (means ± SD). (PDF 14 kb) [file 10565_2017_9394_MOESM4_ESM.pdf]
