# Supplementary material for: Analysis of DNA-damage response to ionizing radiation in serum-shock synchronized human fibroblasts
Source: Cell Biol Toxicol. 2017 May 3;33(4):373–88. doi: 10.1007/s10565-017-9394-9 (PMC5493713; doi:10.1007/s10565-017-9394-9)

a

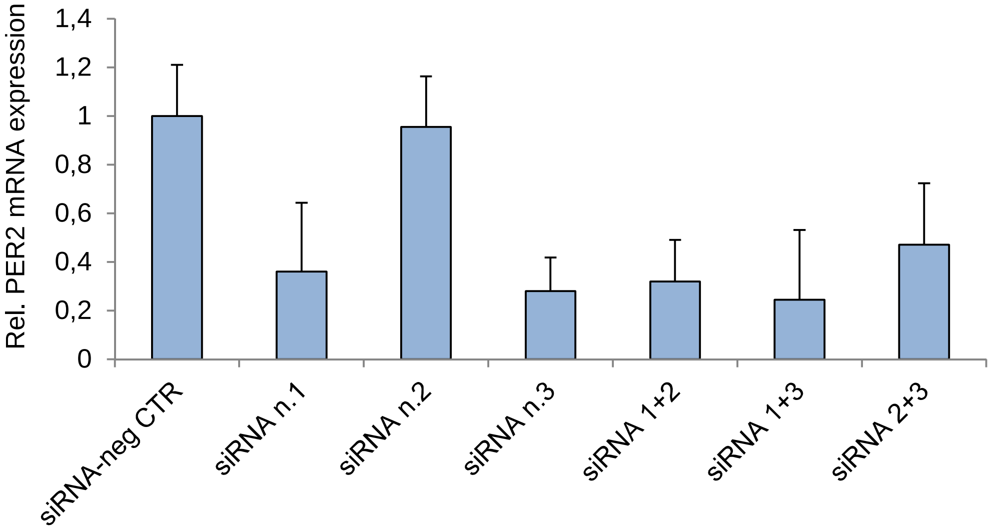

b

siRNA n.1 **PER2HSS113092**

Sequence (5' to 3'): UCCAGUGGACAUGAGACCAACGAAA
Sequence (5' to 3'): UUUCGUUGGUCUCAUGUCCACUGGA

siRNA n.2 **PER2HSS113093**

Sequence (5' to 3'):GAUGUGGGCGUGUUCCACAGUUUCA
Sequence (5' to 3'):UGAAACUGUGGAACACGCCCACAUC

siRNA n.3 **PER2HSS189627**

Sequence (5' to 3'): GCGGCCUCCUAAACCUCCUGCUGAA
Sequence (5' to 3'): UUCAGCAGGAGGUUUAGGAGGCCGC

c

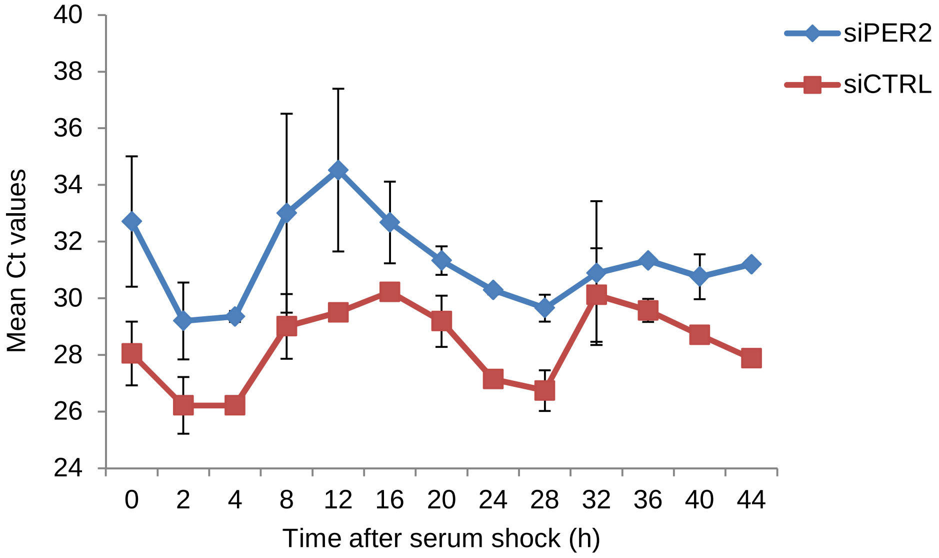

Supplement: Supplementary file 2 — Analysis of PER2 expression by qRT-PCR in human fibroblasts CCD-34Lu transfected with siRNAs. a Relative expression of PER2 transcript in cells transfected with 32 nM of three different Stealth RNAi™ siRNAs delivered alone or in combination, or siCTRL (siRNA with a nonsense/scrambled sequence) using Lipofectamine RNAiMAX. The values (means ± SD) are normalized with GAPDH mRNA as an internal control and plotted as fold-change. b Sequence of three different Stealth RNAi™ siRNAs (PER2HSS113092, PER2HSS113093, PER2HSS189627) tested to silence PER2 gene in CCD-34Lu cells. c Mean Ct (threshold cycle) in CCD-34Lu cells transfected with siPER2n.3 or siCTRL. (PDF 19 kb) [file 10565_2017_9394_MOESM2_ESM.pdf]
